# Does initial buccal crest thickness affect final buccal crest thickness after flapless immediate implant placement and provisionalization: A prospective cone beam computed tomogram cohort study

**DOI:** 10.1111/cid.13060

**Published:** 2022-01-03

**Authors:** Tristan Ariaan Staas, Edith Groenendijk, Ewald Bronkhorst, Luc Verhamme, Gerry Max Raghoebar, Gerrit Jacobus Meijer

**Affiliations:** ^1^ Department of Oral and Maxillofacial Surgery Radboud University Medical Center (RadboudUMC) Nijmegen The Netherlands; ^2^ Department of Preventive and Curative Dentistry Radboud University Medical Center (RadboudUMC) Nijmegen The Netherlands; ^3^ Department of Oral and Maxillofacial Surgery University of Groningen, University Medical Center Groningen Groningen The Netherlands; ^4^ Department of Dentistry Radboud University Medical Center, Radboud UMC Nijmegen The Netherlands

**Keywords:** aesthetic outcome, CBCT analysis, flapless immediate implant placement, immediate restoration

## Abstract

**Background:**

Flapless immediate implant placement and provisionalization (FIIPP) in the aesthetic zone is still controversial. Especially, an initial buccal crest thickness (BCT) of ≤1 mm is thought to be disruptive for the final buccal crest stability jeopardizing the aesthetic outcome.

**Purpose:**

To radiographically assess the BCT and buccal crest height (BCH) after 1 year and to calculate the correlation between initial and final achieved BCT.

**Materials and Methods:**

The study was designed as a prospective study on FIIPP. Only patients were included in whom one maxillary incisor was considered as lost. In six centers, 100 consecutive patients received FIIPP. Implants were placed in a maximal palatal position of the socket, thereby creating a buccal space of at least 2 mm, which was subsequently filled with a bovine bone substitute. Files of preoperative (T0), peroperative (T1) and 1‐year postoperative (T3) cone beam computed tomogram (CBCT) scans were imported into the Maxillim™ software to analyze the changes in BCT‐BCH over time.

**Results:**

Preoperatively, 85% of the cases showed a BCT ≤1 mm, in 25% of the patients also a small buccal defect (≤5 mm) was present. Mean BCT at the level of the implant‐shoulder increased from 0.6 mm at baseline to 3.3 mm immediate postoperatively and compacted to 2.4 mm after 1 year. Mean BCH improved from 0.7 to 3.1 mm peroperatively, and resorbed to 1.7 mm after 1 year. The Pearson correlation of 0.38 between initial and final BCT was significant (*p* = 0.01) and therefore is valued as moderate. If only patients (75%) with an intact alveolus were included in the analysis, still a “moderate correlation” of 0.32 (*p* = 0.01) was calculated.

**Conclusions:**

A “moderate correlation” was shown for the hypothesis that “thinner preoperative BCT's deliver thinner BCT's” 1 year after performing FIIPP.

**What is known**

- Flapless immediate implant placement and provisionalization (FIIPP) in the aesthetic zone may lead to substantial bone loss of the buccal crest, and thereby to retraction of the soft tissues compromising aesthetics, especially in cases of a buccal crest thickness (BCT) ≤1 mm.

**What this study adds**

- By creating a gap between buccal crest and implant surface of at least 2 mm, and by filling this gap with a freeze‐dried bovine bone xenografts, a sufficient BCT can be created to support the soft tissue.
- Even in cases that initially show a thin buccal crest (≤1 mm), 1‐year post‐operatively a sufficient BCT can be achieved. Nevertheless, initial thin (≤0.5 mm) buccal crests, even after FIIPP, may lead to failures.
- Initial BCT matters. The Pearson's correlation was moderate, suggesting that initial thin buccal crests may result in thinner reconstructed buccal crests 1 year post‐operatively.

## 1 INTRODUCTION

Replacement of maxillary incisors by immediate implant placement and provisionalization (IIPP) may be a reliable therapy with respect to implant survival and pink aesthetic outcome.^1^, ^2^, ^3^ This minimal invasive procedure improves patient comfort, reduces both treatment time and postoperative complaints, as well as costs compared to early or delayed placement protocols. However, due to the natural process of post‐extraction bone remodeling,^4^, ^5^, ^6^, ^7^ the pink aesthetic outcome may vary. In this perspective, thickness of the buccal bone crest is crucial.^8^

Dimensional alterations of the facial soft tissues and buccal bone following tooth extraction negatively influence a successful aesthetic outcome in implant therapy.^9^ Cone beam computed tomogram (CBCT) analysis showed vertical loss of the buccal crest up to 7.5 mm within 8 weeks after flapless extraction.^10^ This bone loss compromises the aesthetic outcome, because it is followed by retraction of the covering soft tissues, resulting in midfacial soft tissue recession, which in the end may lead to exposure of the implant surface. Immediately replacing a root by a dental implant itself does not prevent resorption of the buccal crest.^11^, ^12^, ^13^ In order to compensate for bone resorption, bone augmentation procedures in advance of implant installation have been suggested. Ridge preservation procedures, filling the extraction socket with bone or a bone substitute, have proven to be effective in limiting both horizontal and vertical ridge alterations after extraction.^14^, ^15^, ^16^, ^17^ In these procedures, applying freeze‐dried bovine bone xenografts is favorable, as they preserve more alveolar bone volume compared to the use of autogenous bone.^18^, ^19^, ^20^

During immediate implant placement also ridge preservation can be performed, provided that sufficient space is created, which subsequently can be filled with a bone substitute.^21^, ^22^ To create such a gap with optimal dimensions, new insights advocate to install the implant in a more palatal position, on condition of the presence of sufficient apical bone volume. Such a gap leads to less crestal bone resorption and minimal midfacial soft‐tissue recession.^23^, ^24^, ^25^, ^26^, ^27^, ^28^ This buccal gap even allows new bone formation, coronal to the receding buccal bone wall.^29^, ^30^, ^31^ To achieve a minimal gap width of at least 2 mm the use of implants with a smaller diameter is advocated. Thickness of the buccal crest itself also plays an important role, as it consists of bundle bone. As herein only minimal vascularization and regenerative ability are present, thinner buccal alveolar crests will resorb more.^32^ To prevent resorption the buccal gap may be filled with a freeze‐dried bovine bone xenograft. In combination with immediate provisionalization, an instant support of the papillae and midfacial soft tissue is delivered, thereby reducing marginal bone changes.^33^, ^34^, ^35^, ^36^ Only in case of sufficient initial stability provisional restoration is advocated at the time of flapless implant placement,^37^, ^38^, ^39^, ^40^ independent of the patient's biotype.^41^

CBCT data giving insight into the process of buccal bone remodeling after IIPP are rare. This prospective multicenter CBCT study reveals the process of buccal bone remodeling in the aesthetic zone after FIIPP, while placing the implants in a palatal position and simultaneously performing a ridge preservation procedure.

Aim of the study is to evaluate bone remodeling of the reconstructed buccal wall after FIIPP and to inventory if there is relation between “initial buccal crest thickness” on one hand, and the “final thickness” of the reconstructed buccal crest on the other hand. It is hypothesized that the thinner the initial crest thickness, the thinner the reconstructed buccal crest will be after performing FIIPP.

## 2 MATERIAL AND METHODS

### 2.1 Study population

In total, 100 consecutive patients were included in this prospective clinical study between 2014 and 2017. In all patients, one upper tooth was in danger of being lost. This study was approved by the Ethics Committee of the Radboud University Medical Center Nijmegen (2014/157) and registered in the Dutch Trial Register (NTR) on 20 October 2015 (NTR5583/NL4170). Written informed consent to participate in this study, as well as for use and publication of the data, was derived from all participants. This manuscript was written conform the Strengthening the Reporting of Observational studies in Epidemiology (STROBE) guidelines. These guidelines were created to aid the author in ensuring high‐quality presentation of the conducted observational study.

Prerequisites were that the one failing single maxillary incisor was surrounded by two healthy teeth, that the extraction socket was intact and that sufficient occlusal support was present in the absence of periodontal disease and bruxism. Furthermore, to allow for primary implant stability, sufficient apical bone volume had to be present in the apical region.

Besides intact sockets, also sockets showing solely a periapical bone defect or a bone crest defect ≤5 mm, defined as EDS‐2 or EDS‐3,^42^ were included.

Patients suffering from the following habits or diseases were excluded: smoking more than 10 units a day, drug or alcohol abuse, uncontrolled diabetes, pregnancy, or when disturbed bone healing could be expected, such as in case of local or systemic disease, severe osteoporosis, Paget's disease, renal osteodystrophy, radiation in the head–neck region, immune‐suppression or corticosteroids treatment in the recent past. Finally, the aesthetic expectations had to be achievable.

### 2.2 Multicenter

In total, six centers for oral implant therapy participated; one university, one hospital, and four referral dental clinics. In the two first centers, an oral maxillofacial surgeon installed the implants, while the restorative procedure was performed by a separate restorative dentist. In the remaining four centers, the complete IIPP procedure was performed by a dentist trained in oral implantology.

### 2.3 Preoperative measures

Patients were instructed to take 2 g amoxicillin 1 h before surgery, and 500 mg/3× day for 5 days starting in the morning after surgery. If patients were allergic to amoxicillin 600 mg clindamycin 1 h before surgery, and 300 mg/4× day for 5 days starting in the morning after surgery, was advised. In addition, patients had to rinse with 0.12% chlorhexidine solution twice a day for 14 days, starting the day before surgery. Also, it was instructed to take 1 g paracetamol or 600 mg ibuprofen 1 h before surgery.

### 2.4 Operative procedure

**FIGURE 1:**
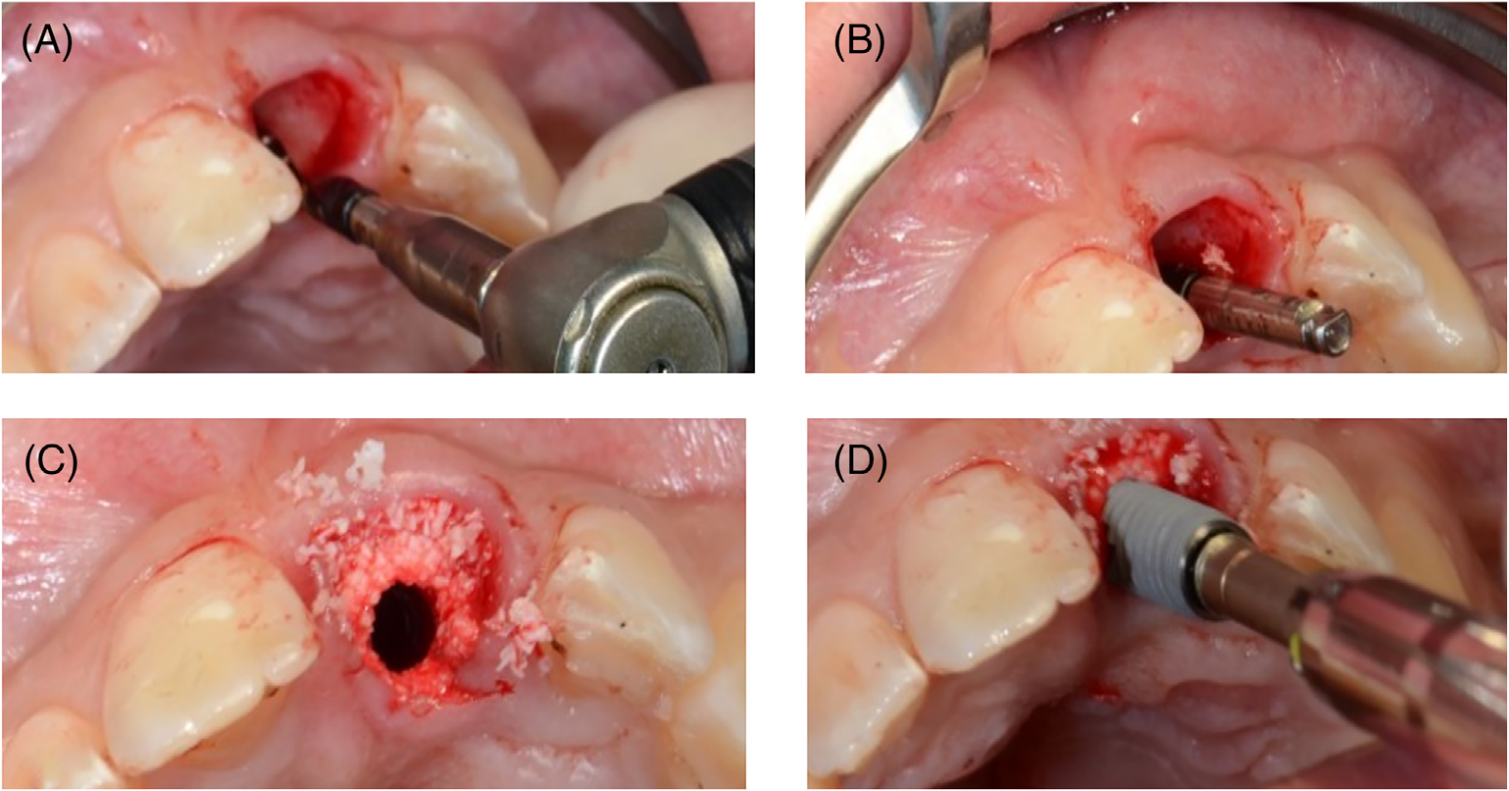
(A) The osteotomy was conducted in the palatal wall, after which (B) the last drill used was placed into the socket. After filling the socket with (C) a mixture of blood and Bio‐Oss™, the drill was removed and (D) the implant installed

After atraumatic tooth removal, an osteotomy was conducted in the palatal wall of the socket in a more palato‐apically direction compared to the original apex (Figure 1A). Subsequently, the last used drill remained in the preparation to prevent bone substitute plugging it (Figure 1B). Hereafter, the socket was filled with a mixture of blood and bovine bone (Bio Oss™ S 0.25–1 mm, Geistlich Biomaterials, Wolhusen, Switzerland) after which the drill was removed carefully by turning it anti‐clockwise, creating a clean corridor to install the implant (NobelActive Conical Connection™ NobelBiocare, Washington, DC) (Figure 1C). The seat of the implant was placed 3 mm apically from the buccal gingival margin and at least 2 mm palatal of the buccal bone plate (Figure 1D) to create the recommended space buccally and to allow a suitable emergence angle of <30°.^43^ To evaluate the implant position a low dose small field CBCT scan was made; in case of inaccuracies still corrections could be conducted.

**FIGURE 2:**
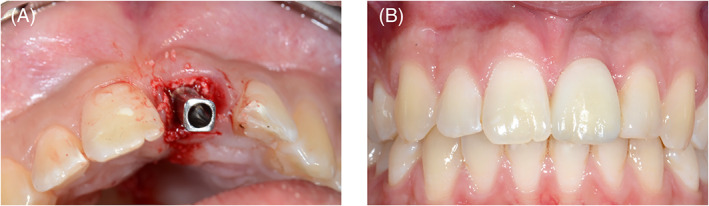
(A) Titanium temporary abutment was placed onto the implant, allowing the fabrication of a provisional crown. (B) The aesthetic result after 1 year

Hereafter, a titanium temporary customized platform‐switch Procera™ abutment (Nobel Biocare, Washington, DC) was placed (Figure 2A) allowing fabrication of a composite screw‐retained provisional restoration. Care was taken to prevent contact with the antagonistic dentition in occlusion or articulation. After implant placement (3–9 months), the final impression was taken to fabricate either an individualized, screw‐retained, zirconium‐oxide porcelain veneered crown, or an individualized zirconium‐oxide abutment (Procera™, NobelBiocare, Washington, DC) with a resin cemented porcelain facing (Figure 2B).^44^

### 2.5 Radiographic procedure measurements

To minimize the effective dose, only small field‐of‐view scans (6 × 6 cm) were applied. For analysis, the preoperative, peroperative, and CBCT data after 12 months were imported as Digital Imaging and Communications in Medicine (DICOM) files into the Maxillim™ software (version 2.3.0.3, Medicim NV, Mechelen, Belgium).

**FIGURE 3:**
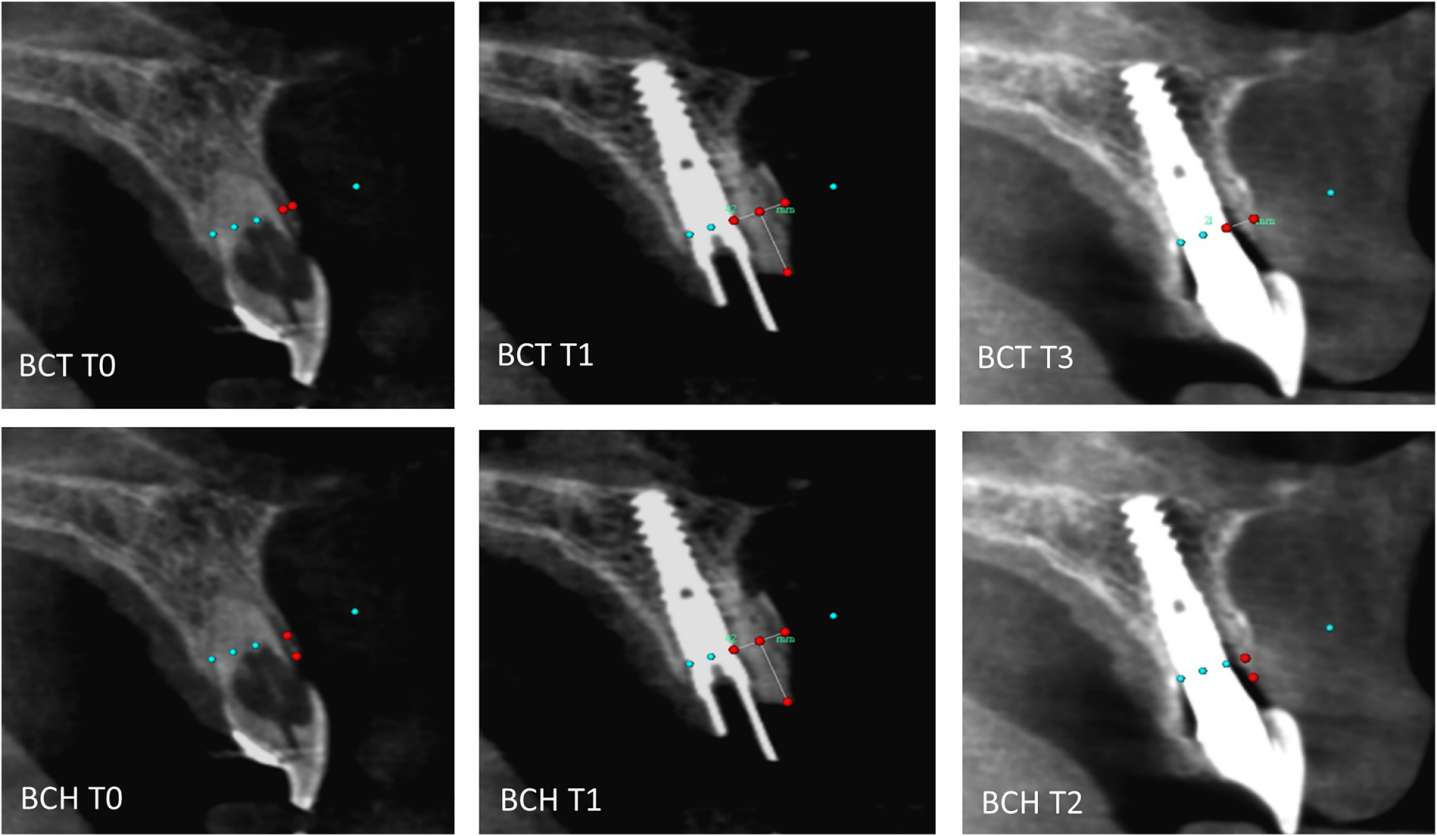
BCT: bone crest thickness. BCH: bone crest height. Preoperatively (T0), direct postoperatively (T1), and after 1 year (T3) measurements (red dots) were conducted. The green dotted reference line reflects the shoulder of the implant

Superimposition of the different CBCT scans using the voxel‐based alignment procedure in Maxilim™ scans was performed prior to analysis of the buccal crest. Using both the palate, anterior nasal spina, and adjacent teeth as reference area for voxel‐based alignment, optimal superimposition of the dimensions of the (reconstructed) buccal crest became feasible. Thickness of the buccal crest was measured at the level of the implant‐shoulder, ensuring that thickness of the buccal crest was measured at the same position and angulation at all time points. By subtracting the preoperative (T0), peroperative (T1), and 1 year postoperative (T3) dimensions, both changes in buccal crest thickness (BCT) and buccal crest height (BCH) could be calculated (Figure 3).

Buccal crest thickness (BCT‐T0) before treatment was measured using two methods: (1) directly or (2) by subtracting the distance from the inner buccal crest (IBC) to the implant from the outer buccal crest (OBC) to the implant.

Between midfacial, 1 mm to the mesial or 1 mm to the distal side, no difference in measurements for thickness or height is present,^36^ therefore, solely midfacial measurements were conducted.

### 2.6 Statistical methods

For all measurements, the range, median, mean and standard deviation were calculated. Differences in BCT and BCH were tested with a paired sample *t*‐test. Statistics were calculated for all clinical parameters using SPSS (SPSS Inc., Chicago, IL). Statistical significance was defined as *p* ≤ 0.05.

The inter‐observer performance was analyzed using a paired sample *t*‐test. For this purpose in total 36 measurements were repeated. The reliability was calculated as Pearson's correlation, and the random error was calculated as the standard deviation of the difference between observers, divided by √2.

To assess if the two methods (BCT vs OBC minus IBC) led to a different outcome also a paired sample *t*‐test was conducted.

To inventory if there was a correlation between the “initial BCT” and “final BCT after 1 year,” the Pearson's correlation coefficient was calculated.

## 3 RESULTS

Of the 100 included patients, 98 (57 females, 41 males) were available for evaluation. One patient was excluded because of trauma resulting in implant loss, another moved abroad. Age of the included patients varied between 17 and 80 years with an average of 45.8 years.

On average, IIPP took place 37 days (range 0–210 days) after intake. Reasons for extraction were trauma, root fracture, failed endodontic treatment, or lack of ferrule.

NobelActive™ CC implants with a diameter of 3.0 mm (6×) and 3.5 mm (17×) implants were used to replace lateral incisors. Central incisors were replaced by NobelActive™ CC with 3.5 mm diameter (30×) or with 4.3 diameter (45×). Implant length varied between 11.5 and 18 mm. In all cases, primary implant stability was sufficient to allow immediate provisional restoration. No implant was lost during the first year of the study; all implants received a final restoration.

### 3.1 CBCT‐analysis

In 17 cases, one of the CBCT‐scans (T0, T1, or T2) could not be interpreted due to movement artifacts, scattering, or beam hardening. In total, 81 complete CBCT series were analyzed.

Reliability was 0.901 (*p* < 0.001), showing a satisfactory correlation between both observers. There was no evidence of a structural difference between both observers as the mean difference was 0.069 mm, with 95% CI = [−0.037–0.175 mm] (*p* = 0.192). The random error was 0.222 mm.

**FIGURE 4:**
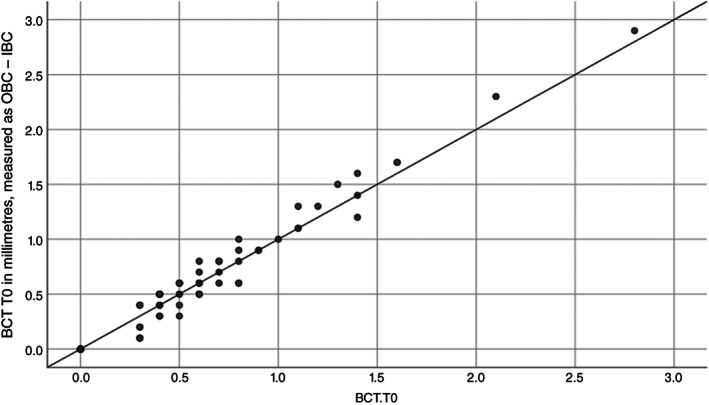
Two methods to measure buccal bone thickness (BCT). The x‐axis represents the BCT which was directly measured. The y‐axis reflects the BCT measured as OBC‐IBC: the difference between the “outer buccal crest” (OBC) and the “inner buccal crest” (IBC) in relation to the implant surface

Initial BCT‐T0 was measured (1) directly or (2) by subtracting OBC from IBC. The paired sample correlation was 0.984. Between used methods, the measured difference in BCT was 0.01 mm (*p* = 0.241). Therefore, both methods are valid for measuring the BCT (Figure 4).

Both mean BCT and BCH per time point are depicted in Table 1. Direct post‐operatively (T1), mean BCT increased from 0.6 mm at baseline (SD = 0.5) to 3.3 mm (SD = 1.2). After 1 year (T3) mean BCT reduced to 2.4 mm (SD = 1.1).

**TABLE 1:** Mean and standard deviation of the bone crest thickness (BCT) and bone crest height (BCH) on three time points (T0, T1, T2)

	Minimum	Maximum	Mean (mm)	Standard deviation
Bone cortical thickness				
BCT‐T0	0.0	2.8	0.6	0.5
BCT‐T1	1.6	8.1	3.3	1.2
BCT‐T3	0.0	5.0	2.4	1.1
Bone cortical height				
BCH‐T0	−7.5	5.9	0.7	2.1
BCH‐T1	0.0	5.7	3.1	1.2
BCH‐T3	−12.3	4.9	1.7	2.4

Mean BCH at T0 was 0.7 mm (SD = 0.5), which enlarged to 3.1 mm (SD = 1.2) direct postoperatively (T1). Over a period of 1 year (T3) BCH condensed to 1.7 mm (SD = 2.4).

With respect to BCT and BCH, differences between T1 versus T0, T3 versus T0, and T3 versus T1 were statistically significant (all *p* = 0.003) (Table 2).

**TABLE 2:** Differences in bone crest thickness (BCT) and bone crest height (BCH) are significant between time points T1‐T0, T2‐T0, and T3‐T2

	Mean (mm)	Standard deviation	Significance (2‐tailed)
Difference in bone cortical thickness			
BCT‐T1 minus BCT‐T0	2.7	1.0	<0.001
BCT‐T3 minus BCT‐T0	1.9	1.0	<0.001
BCT‐T3 minus BCT‐T1	−0.8	1.0	<0.001
Difference in bone cortical height			
BCH‐T1 minus BCH‐T0	2.4	2.1	<0.001
BCH‐T3 minus BCH‐T0	0.9	2.6	<0.003
BCH‐T3 minus BCH‐T1	−1.5	2.1	<0.001

Preoperatively, 85% of the patients presented a BCT‐T0 of ≤1 mm (Table 3). At T1, thus immediately after performing IIPP, 98% of all patients showed a BCT‐T1 of at least 2 mm (Table 3). After 1 year, 8 patients (10%) showed a BCT‐T3 less than 1 mm (Table 3): 2 patients (2.5%) showed a BCT‐T3 of 0.6 and 0.8 mm each. In the other 6 (7.5%) patients, no bone crest was present (BCT‐T3 = 0). In these 6 patients also BCH‐T3 failed, meaning that after 1 year, bone height was lower than the level of the implant‐shoulder.

**TABLE 3:** Distribution of patients in relation to their BCT at times T0, T1, and T3

BCT‐T0		BCT‐T1		BCT‐T3	
=0.0 mm	25%			=0.0 mm	7.5%
0.0–1.0 mm	60%			0.0–1.0 mm	2.5%
≥1.0 mm	13%	1.0–2.0 mm	2%	1.0–2.0 mm	17%
≥2.0 mm	2%	≥2.0 mm	98%	≥2.0 mm	73%

**FIGURE 5:**
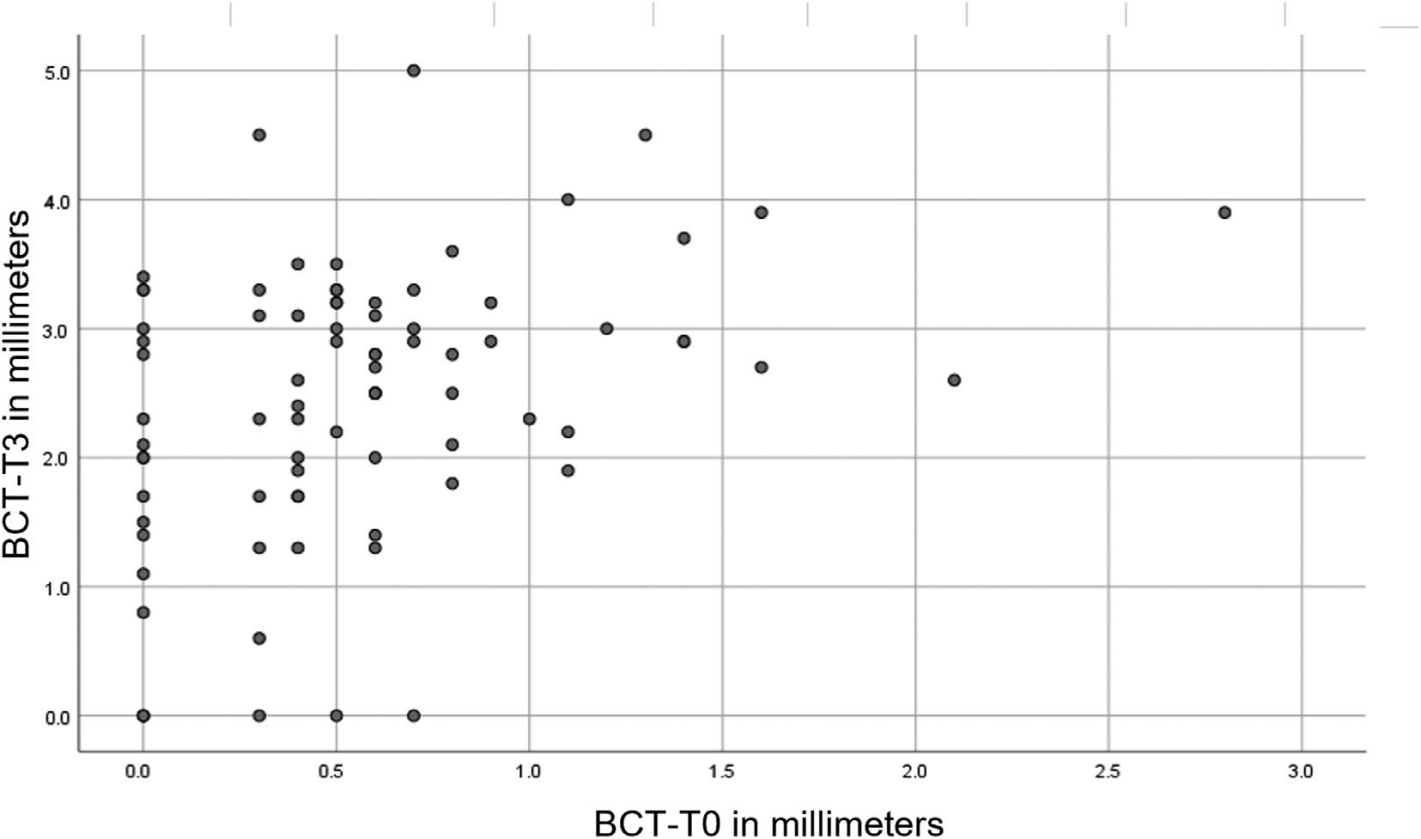
In this graph, the Pearson's relation between the initial BCT‐T0 (x‐axis) and the final achieved BCT‐T3 (y‐axis) is depicted for all 81 patients

To assess how the initial bone crest thickness (BCT‐T0) is related to the final bone crest thickness (BCT‐T3), the Pearson's correlation was calculated for all 81 patients (Figure 5). The outcome of 0.38 (*p* = 0.01) suggests that a moderate correlation is present. For solely the 61 patients (75%: Table 3) with an intact alveolus at T0 a Pearson's correlation of 0.32 (*p*: 0.011) was calculated, which still stands for a moderate correlation.

## 4 DISCUSSION

Although IIPP procedures are more accepted nowadays, midfacial recession as a result of bone loss of the buccal crest is still considered to be a major risk. Publications about this topic are difficult to compare to each other, because surgical procedures differ substantially with respect to the choice of implant type, implant diameter, implant position, and “to raise a flap or not.” Furthermore, discussion is ongoing if additional connective tissue grafts or bone substitutes should be applied. Confusing is also that in most publications, with respect to the prosthetic treatment, various materials and shapes of abutment were included. Moreover, debate continues if, and at what time point, provisional restorations should be used.^45^

From a patient's point of view, immediate tooth replacement is an attractive strategy, because in one session both aesthetics and comfort are delivered together with a substantial gain in treatment time, as in the same treatment session both the tooth is extracted and the implant installed. Also with respect to the aesthetic result after 1 year IIPP offers advantages: midfacial recession is 0.75 mm less compared to delayed restoration after 1 year.^46^

With respect to the question if “whether or not” a buccal flap should be raised, Naji et al. presented the 6 months' results of a CBCT study in which different soft tissue techniques were compared.^47^ In three groups of each 16 patients, a buccal gap of at least 2 mm was created: group 1 received a bone graft and membrane, after which the wound bed was closed with a primary flap. Group 2 received primary flap closure only. In group 3, no extra technique was applied; solely immediate implant installation was conducted. The least horizontal dimensional change after implant placement was recorded for group 3, in which also the least postoperative pain was monitored. Both can be explained by the fact that no flap was raised, thereby stressing the importance of the local blood supply and, as such, the vitality of the soft tissues. These results are in agreement with others, which confirmed that, if no flap is elevated, a greater preservation of the buccal alveolar bone width from resorption was seen.^48^, ^49^

Considering the need of filling the gap, Naji et al. suggested that solely a thick bone crest with a BCT of ≥1 mm, allows a stabilized formed coagulum without the need for regenerative materials.^47^ Others stated that in case of an initial buccal bone plate width of <1 mm or with fenestration, gap grafting, and regeneration are recommended to enhance bone filling and reduce the bone reduction.^50^, ^51^, ^52^

CBCT is a useful tool that has been successfully used for reproducibility and accuracy of bone crest level measurements,^53^, ^54^ as corroborated in the present study showing a mean difference of 0.069 mm between both observers (*p* = 0.192).

The initial mean width BCT in our study was 0.6 mm (SD 0.5) which is in accordance with previous studies using CBCT scans to measure bone width around maxillary anterior teeth.^8^, ^55^, ^56^ With our IIPP protocol immediately after surgery BCT a gain in thickness of 2.7 mm was achieved: from 0.6 mm (mean) to 3.3 mm (mean). After 12 months, a decrease of 0.9 mm was observed still leaving a BCT of 2.4 mm (mean). In only a few articles, both initial and postoperative BCT were measured in combination with IIPP. Although Morimoto et al. described 12 patients retrospectively and also filled the buccal gap with bone graft material, they did not create a buccal gap of at least 2 mm.^55^ They reported an initial median BCT of 0.5 mm, and a median thickness of 1.8 mm after 1 year, resulting in a total gain of 1.3 mm, which is lower as reported in our study (1.8 mm) when generating a minimal gap of 2 mm. Degidi et al. created buccal gaps of between 1 and 4 mm and filled the buccal gaps with Bio‐Oss™ Collagen (Geistlich Pharma AB, Wolhusen, Switzerland). Although they did not present an initial BCT, the same decrease of 0.9 mm in the mean BCT was reported after 1 year: from 3.0 to 2.1 mm.^57^

Unfortunately, also with respect to the vertical dimension, Degidi et al. presented no initial bone heights, and thereby no initial increase in BCH. The immediate postoperatively achieved BCH of 3.0 mm reduced to 2.2 mm after 1 year. This reduction (0.8 mm) is less than the 1.4 mm in our CBCT study, in which BCH decreased from 3.1 to 1.7 mm.^57^ However, this can be explained by the composition of their patient population: 50% of the implant sites were not in the front, but in the premolar and the canine region.^57^ Furthermore, only patients were introduced if the BCT was at least 0.5 mm, while in our study also cases with a smaller BCT were allowed. Morimoto et al. only presented a preoperative BCH (median 1.5 mm) and after 1 year (median 1.1 mm). It is unclear if their clinical procedure resulted in a gain in BCH.^55^

The vertical increase in BCH, as measured in this study, may seem surprising, but is in agreement with the gain of height that already was reported in the ridge preservation studies from Iasella et al. in 2003^14^ and Vance et al. in 2004^58^ who reported an average gain of 1.3 and 0.7 mm, respectively.

Applying a bone substitute simultaneously with IIPP significantly enlarged the buccal crest both in width and height. Key question is the exact composition of the final buccal crest. After all, a limitation of this study is that no histology of the buccal bone volume was conducted; it was not specified which percentage of buccal crest consists of newly formed bone or bone substitute. It can be secured that immediately after application of the bone substitute the buccal bone crest consists of a combination of the original buccal bone at the outside and bone substitute at the inside. The measured horizontal bone reduction after 12 months can be explained by resorption of the buccal bundle bone initiated by the removal of the periodontal ligament, reducing local blood supply and its regenerative capacity. Horizontal reduction of BCT also can be clarified by condensation of the bone substitute particles in time.

In 20 (25%) of the patients preoperatively, a small buccal bone defect (≤5 mm) was present. If this group was included in the statistical analysis, the Pearson correlation between initial and final BCT was 0.38. If this group was excluded from analysis, still a significant Pearson's correlation of 0.32 remained. As such there is indeed a moderate correlation indicating that thin buccal plates will result also in thinner buccal plates after reconstruction. Of course, also other factors will influence the end result after reconstruction, such as age, general condition, and width of the created gap.

To our knowledge, this is the first prospective study assessing the relation between the initial BCT and the BCT after 1 year. A ‘moderate correlation’ was shown for the hypothesis that ‘thinner preoperative BCT's deliver thinner BCT's’ one year after performing FIIPP. Nevertheless, independent of the initial BCT, 1 year after following the presented FIIPP protocol, both bone crest thickness and height were in 90% of the cases still substantial, meaning that more than the required minimal BCT of 1 mm was present, thereby creating a stable base for the soft tissues.

The lesson to be learned is that FIIPP may be successful in cases of a bone defect at the implant shoulder (BCT‐T0 = 0); however, it is not a panacea. In total, 20 of such cases (25%) were included: 14 were successful (BCT‐T3 ≥1 mm) and 6 cases (7.5%) failed, meaning that the bone crest both in thickness and height scored zero 1 year postoperatively.

Long‐term prospective studies need to be performed to prove if both BCT and BCH will also be stable over time. Retrospective CBCT‐data after 7 years showed already promising results.^59^

## CONFLICT OF INTEREST

The authors declare no conflict of interest.

## AUTHOR CONTRIBUTIONS

Tristan Ariaan Staas conceptualized the project idea, conducted the literature search, collected and analyzed the data, and drafted the manuscript. Edith Groenendijk conceptualized the project idea, collected and analyzed data, and made corrections to the drafted manuscript. Gerry Max Raghoebar contributed to the data analysis. Ewald Bronkhorst conducted the statistical analysis. Gerry Max Raghoebar and Gert Jacobus Meijer critically reviewed and revised the manuscript.

## Data Availability

The data that support the findings of this study are available on request from the corresponding author. The data are not publicly available due to privacy or ethical restrictions.
